# Impact of blood count recovery on outcomes of acute myeloid leukemia patients achieving morphologic leukemia-free state

**DOI:** 10.1038/s41408-018-0094-3

**Published:** 2018-06-07

**Authors:** Wen-Yan Cheng, Yong-Mei Zhu, Zhao Liu, Xiang-Qin Weng, Jing-Ni Sui, Yun-Shuo Chen, Shi-Yang Wang, Yang Shen

**Affiliations:** Department of Hematology, Shanghai Institute of Hematology, Ruijin Hospital Affiliated to Shanghai Jiao Tong University School of Medicine, Shanghai, China

Acute myeloid leukemia (AML) is a group of heterogeneous hematological malignancies. Clinical features such as age, performance status, and cytogenetic and molecular abnormalities at diagnosis are all pre-treatment prognostic factors. In recent years, post-treatment parameters including minimal residual disease (MRD) and blood count recovery at the time of attaining morphologic leukemia-free state (MLFS) have drawn much research interest and considered to be significant in predicting the risk of relapse and survival. The concept of complete remission with incomplete hematologic recovery (CRi), fulfilling all CR criteria except for absolute neutrophil count <1000 per μL or platelet count <100,000 per μL in peripheral blood, was first proposed by Cheson et al.^1^, who suggested that patients with CRi had a relatively poor outcome compared with CR especially during initial chemotherapy. Subsequently, Larson et al.^2^ reported a significantly better relapse-free survival (RFS) rate in patients with CR than CR with incomplete platelet recovery (CRp, CR except for platelet count <100,000 per μL) who did not receive further treatment beyond gemtuzumab ozogamicin. In a large series^3^, achievement of CRp rather than CR was independently associated with an inferior RFS. The long-term survival of CRi and its correlation with other clinical parameters, especially cytogenetic and molecular abnormalities and MRD, remain to be explored. Here we report a study of patients with de novo AML who achieved CR or CRi after the first two cycles of chemotherapy and make comparisons between the two groups.

From January 2014 through December 2016, 350 consecutive newly diagnosed non-M3 adult AML patients were enrolled in this study. Patients with AML secondary to antecedent hematologic disorders were excluded. Cytogenetic risk categories were defined according to NCCN guidelines^4^. Younger patients (age ≤60) and fit elderly patients received standard first-line “3 + 7” induction regimens, and high-dose cytarabine-based consolidation therapy. Unfit patients underwent either low-dose first-line regimens or other low-intensity therapy. Patients who met the standards of MLFS but failed to acquire neutrophil or platelet recovery after induction or first cycle of consolidation therapy were classified into CRi group. This study was approved by the ethic committee of Ruijin hospital. All patients had given informed consent according to the Declaration of Helsinki.

Gene mutations/fusions including *FLT3*-ITD and -TKD, *C-KIT*, *N-RAS*, *CEBPA*, *DNMT3A*, *NPM1*, *MLL*, *IDH2*, *AML1-ETO*, and *CBFβ-MYH11* were detected as previously reported^5^. Detection of MRD was based on leukemia-associated immunophenotype (LAIP) at diagnosis and performed by using 10-color multiparametric flow cytometry.

Kaplan–Meier method was used to calculate the distribution of overall survival (OS) and RFS. Cox proportional hazards model was applied for multivariate analysis of OS and RFS, and variables with *P* value below 0.20 in univariate analysis were chosen to form the final model.

Clinical characteristics of the 350 patients are summarized (Supplementary Table 1). Among which, 26 patients died early, and 14 patients lost contact after induction therapy. Among the remaining 310 patients whose responses were evaluable, 230 patients (74.2%) achieved either CR (*n* = 179) or CRi (*n* = 51), 57 patients (18.4%) and 23 patients (7.4%) showed no remission (NR) and partial remission (PR), respectively. Further analyses were mainy conducted in the CR and CRi cohorts. At diagnosis, age, white blood count, hemoglobin level, and bone marrow (BM) blasts were similar between the two groups. Principal difference was the lower platelet count in CRi patients than those with CR (median, 29 × 10^9^/L vs. 49 × 10^9^/L, *P* = 0.024). Patients achieving CRi were prone to having less favorable cytogenetics (8% vs. 19.4%, *P* = 0.06), which was approximate to those with PR or NR (8.2%). Forty-eight patients (20.9%) with CR or CRi underwent hematopoietic cell transplantation (HCT) at least after two courses of treatment.

In comparison to patients with CR, patients in CRi group had a lower incidence of *C-KIT* mutations (4% vs. 14.9%, *P* = 0.039) and a higher frequency of biallelic *CEBPA* mutations (bi*CEBPA*, 33.3% vs. 19.6%, *P* = 0.038). We then classified gene abnormalities into three subgroups per 2017 ELN recommendations^6^, and found that the percentages of favorable, intermediate, and adverse genetic alterations were similar among CR and CRi group (Supplementary Table 2).

A total of 196 patients (85.2%) with CR or CRi presented definite LAIP and had MRD results after induction therapy. Patients with CRi had a significantly higher MRD level compared with those with CR (median, 0.046% vs. 0.017%; *P* = 0.007). Similarly, a higher frequency of positive MRD (MRD ≥0.1%) could be seen in patients with CRi (45.2% vs. 22.7%, *P* = 0.004) (Supplementary Table 3).

Patients in CR group had a longer RFS compared with those whose best response was CRi (*P* = 0.045, Fig. 1a), with an estimated median RFS of 33 and 18 months, respectively. Similarly, patients with CR had a significantly better OS than CRi (*P* = 0.001, Fig. 1b). The estimated median OS was 37 months in patients with CRi while it was “not reached” in those with CR. As presented in Fig. 1c–f, for patients who did not receive HCT, failure in achieving blood count recovery conferred a shorter RFS (median RFS, 31 months for CR, 18 months for CRi; *P* = 0.044) and OS (median OS, “not reached” for CR, 23 months for CRi; *P* < 0.001). While for those who underwent HCT, including 9 patients (17.6%) with CRi and 39 patients (21.8%) with CR, the negative effect of CRi on both RFS (median RFS, 35 months for CR, “not reached” for CRi; *P* = 0.815) and OS (median OS, “not reached” for both groups; *P* = 0.752) was greatly attenuated.

**Fig. 1:**
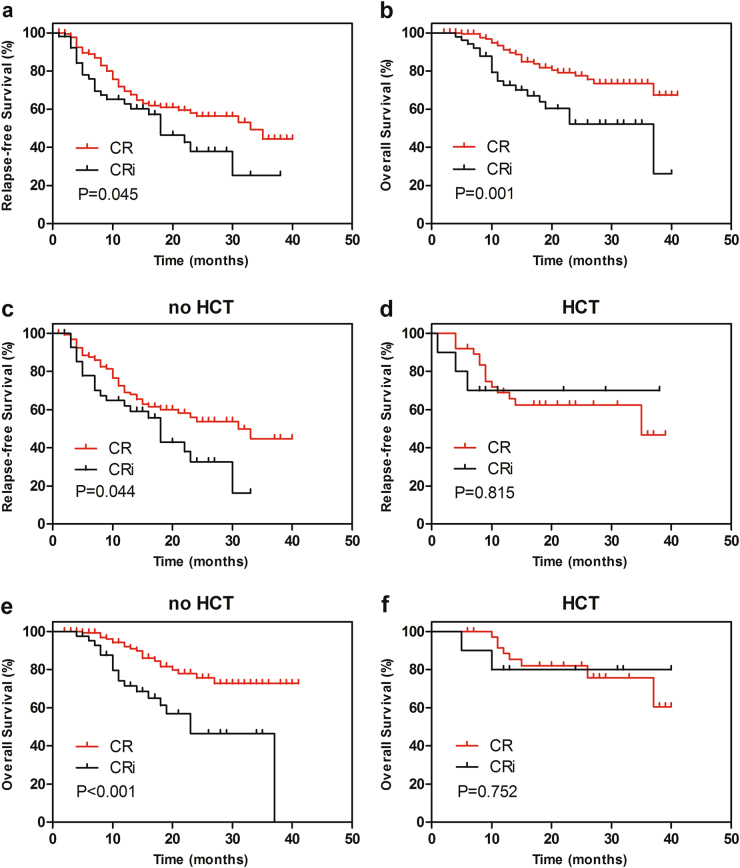
Kaplan–Meier curves for probability of relapse-free and overall survival. **a** Relapse-free survival (RFS) for patients who achieved complete remission (CR) or CR with incomplete hematologic recovery (CRi). **b** Overall survival (OS) for patients who achieved CR or CRi. **c**, **e** RFS and OS for patients with CR or CRi who did not receive HCT. **d**, **f** RFS and OS for patients with CR or CRi who received HCT

We conducted univariate and multivariate analyses for RFS and OS (Table 1). After accounting for covariates, achievement of CRi other than CR was an independent factor associated with shorter RFS (hazard ratio (HR), 1.869; 95% confidence interval (CI), 1.087–3.213; *P* = 0.024) and OS (HR, 3.257; 95% CI, 1.624–6.530; *P* = 0.001). Age, *MLL*-fusion genes, *MLL*-PTD, and *C-KIT* mutations were also significant factors related to prognosis.

**Table 1:** Univariate and multivariate analyses for relapse-free and overall survival

Factor	RFS				OS			
	Univariate		Multivariate		Univariate		Multivariate	
	HR (95% CI)	*P* value	HR (95% CI)	*P* value	HR (95% CI)	*P* value	HR (95% CI)	*P* value
Age	1.026 (1.012–1.041)	<0.001	1.022 (1.005–1.039)	0.010	1.044 (1.024–1.065)	<0.001	1.051 (1.025–1.077)	<0.001
WBC (×10^9^/L, >30 vs. ≤30)	1.583 (1.025–2.445)	0.038		NS	1.070 (0.581–1.970)	0.829		
Cytogenetics								
Intermediate vs. favorable	0.754 (0.430–1.322)	0.324			0.890 (0.421–1.881)	0.760		
Unfavorable vs. favorable	1.241 (0.530–2.906)	0.620			1.881 (0.667–5.303)	0.232		
CRi vs. CR	1.582 (0.999–2.507)	0.051	1.869 (1.087–3.213)	0.024	2.546 (1.439–4.505)	0.001	3.257 (1.624–6.530)	0.001
MRD (positive vs. negative)	1.411 (0.866–2.297)	0.167		NS	1.970 (1.032–3.761)	0.040		NS
*FLT3-*ITD	1.773 (1.000–3.143)	0.050		NS	1.300 (0.581–2.911)	0.523		
*NPM1*m^+^/*FLT3-*ITDm^−^	0.834 (0.471–1.478)	0.534			1.015 (0.492–2.092)	0.968		
*MLL*-fusion gene	3.083 (1.223–7.705)	0.016	3.514 (1.045–11.815)	0.042	1.863 (0.447–7.760)	0.393		
*MLL-*PTD	2.456 (0.990–6.096)	0.053	2.937 (1.039–8.303)	0.042	3.623 (1.290–10.174)	0.015	5.360 (1.569–18.319)	0.007
*C-KIT*	1.508 (0.849–2.678)	0.161	2.555 (1.365–4.782)	0.003	1.670 (0.807–3.454)	0.167	3.670 (1.626–8.283)	0.002
Biallelic *CEBPA*	0.636 (0.364–1.109)	0.111		NS	0.465 (0.198–1.092)	0.079		NS
HCT	0.803 (0.472–1.366)	0.419			0.761 (0.379–1.530)	0.444		

*RFS* relapse-free survival, *OS* overall survival, *HR* hazard ratio, *CI* confidence interval, *WBC* white blood cell, *CR* complete remission, *CRi* complete remission with incomplete hematologic recovery, *MRD* minimal residual disease, *HCT* hematopoietic cell transplantation

Prior reported data demonstrated CRi or CRp as an independent factor related to shorter duration of RFS, but less relavant to OS^2,3^. In other cases, however, CRi lost prognostic value in multivariate analysis^7,8^. Our study indicated that even when other recognized prognostic factors were considered, CRi was independently associated with inferior RFS and OS. Two aspects may underlie the relatively poor outcomes of CRi. Firstly, the frequency of favorable cytogenetics was lower in patients with CRi than those with CR, which may reflect the importance of cytogenetic risk in determining the depth of remission. Moreover, MRD level after therapy is a well-recognized prognostic factor in both acute lymphoblastic leukemia and AML^8,9^. We observed that patients with CRi were more likely to have a higher MRD level and greater frequency of MRD positive status than those with CR after initial induction, which was also observed by Chen et al.^10^ who revealed a strong correlation between MRD and response. We speculate that level of blood count at remission may reflect the amount of minimal residual leukemic cells, which may disrupt hematopoietic regeneration by their toxicity to normal progenitor cells. However, the unfavorable prognostic effect of CRi was greatly attenuated among patients whose MRD was eliminated through HCT, which was similar to the results of the study by Chen et al.^10^ These findings suggest that in combination of blood count and MRD at remission, we are probable to identify patients at a higher risk of relapse and prevent it by using more intensive chemotherapy or selecting novel therapeutic agents to elimilate residual leukemia cells and following with HCT, which may improve the outcome of patients with CRi and could be a direction of future clinical trials.

We previously reported that *CEBPA* mutations accounted for 22% of cytogenetically normal AML (CN-AML) in a large series^5^, a propotion that was higher compared with 12.8% in a 1182 CN-AML cohort^11^, which may reflect the difference in genetic backgrounds between Chinese patients and their European counterparts. However, the long-term prognosis of bi*CEBPA* in our cohort (3-year OS rate, 57%)^5^ was not as good as the results of the study by Taskesen et al.^11^ (5-year OS rate, 63%), which might be partially because that patients with bi*CEBPA* in our cohort were more likely to achieve CRi. Notably, patients with bi*CEBPA* in this study presented a lower platelet count at diagnosis compared with those without (median, 24 × 10^9^/L vs. 49 × 10^9^/L, *P* < 0.001; Supplementary Table 4), which was also observed by Taskesen et al.^11^ These results suggest that *CEBPA* mutations may exert adverse impact on hematopoietic differentiation and recovery of blood count after chemotherapy. In contrast, although we^12^ and others^13^ have reported that *C-KIT* mutations were adverse events in core binding factor leukemias, they were less common in CRi patients compared with those who achieved CR, which needs to be verified in large population.

In summary, our study demonstrates that achieving CRi rather than CR after early cycles of chemotherapy independently predicts shorter RFS and OS. Monitoring the quality of remission through blood count is a straightforward and convenient way to assess the risk of relapse and survival, which should be considered in therapeutic decision-making after remission.

## Electronic supplementary material

Supplementary information
