# Supplementary material for: Impact of blood count recovery on outcomes of acute myeloid leukemia patients achieving morphologic leukemia-free state
Source: Blood Cancer J. 2018 Jun 7;8(6):53. doi: 10.1038/s41408-018-0094-3 (PMC6002522; doi:10.1038/s41408-018-0094-3)
Supplement: Supplementary file 1 — Supplementary information [file 41408_2018_94_MOESM1_ESM.docx]

The supplementary information includes four tables. Supplementary Table 1 shows clinical characteristics of whole patients. Supplementary Table 2 and 3 demonstrates the association of peripheral blood recovery with gene abnormalities and MRD level, respectively, and supplementary Table 4 shows the relationship between biallelic *CEBPA* mutations and platelet count at diagnosis.

**Supplementary Table 1.** Clinical characteristics

| Factor | Overall cohort  (N=350) | NR or PR  (N=80) | CR  (N=179) | CRi  (N=51) | P value  (CR vs. CRi) |
| --- | --- | --- | --- | --- | --- |
| Age (y)  Median  Range | 46  18-77 | 52  18-73 | 43  18-77 | 46  18-71 | 0.209 |
| Male gender, n (%) | 174 (49.7) | 38 (47.5) | 93 (52) | 25 (49) | 0.711 |
| WBC count, × 10^9^/L  Median  Range | 14.8  0.8-419.9 | 21  1-287.7 | 14.3  1-419.9 | 9  0.8-139.7 | 0.14 |
| Hemoglobin, g/dL  Median  Range | 86  30-162 | 81  45-143 | 86  30-162 | 91  32-150 | 0.272 |
| Platelet count, × 10^9^/L  Median  Range | 42  2-1726 | 53  3.7-447 | 49  4-1726 | 29  5-512 | 0.024 |
| Bone marrow blasts, %  Median  Range | 67  14-98 | 71  20-95 | 66  20-98 | 61  18.5-97.5 | 0.364 |
| Cytogenetics  Favorable, n (%)  Intermediate, n (%)  Unfavorable, n (%)  Missing/unknown | 43 (13.6)  233 (73.7)  40 (12.7)  34 | 6 (8.2)  50 (68.5)  17 (23.3)  7 | 31 (19.4)  116 (72.5)  13 (8.1)  19 | 4 (8)  40 (80)  6 (12)  1 | 0.06  0.29  0.581 |
| HCT  Yes, n (%)  No, n (%) | 68 (19.4)  282 (80.6) | 20 (25)  60 (75) | 39 (21.8)  140 (78.2) | 9 (17.6)  42 (82.4) | 0.521 |

Abbreviations: NR, no remission; PR, partial remission; CR, complete remission; CRi, complete remission with incomplete hematologic recovery; WBC, white blood cell; HCT, hematopoietic cell transplantation.

**Supplementary Table 2.** Correlation between gene abnormalities and response

| Gene Abnormality | Overall cohort (N=350) | NR or PR  (N=80) | CR  (N=179) | CRi  (N=51) | P value  (CR vs. CRi) |
| --- | --- | --- | --- | --- | --- |
| *AML1-ETO*  Positive, n (%)  Negative, n (%) | 49 (14)  301 (86) | 9 (11.3)  71 (88.8) | 31 (17.3)  148 (82.7) | 5 (9.8)  46 (90.2) | 0.193 |
| *CBFβ-MYH11*  Positive, n (%)  Negative, n (%) | 24 (6.9)  325 (93.1) | 0 (0)  80 (100) | 15 (8.4)  163 (91.6) | 4 (7.8)  47 (92.2) | 1 |
| *FLT3-*ITD  Mutated, n (%)  Unmutated, n (%) | 52 (14.9)  298 (85.1) | 19 (23.8)  61 (76.3) | 21 (11.7)  158 (88.3) | 4 (7.8)  47 (92.2) | 0.431 |
| *FLT3-*TKD  Mutated, n (%)  Unmutated, n (%) | 17 (4.9)  333 (95.1) | 4 (5)  76 (95) | 10 (5.6)  169 (94.4) | 2 (3.9)  49 (96.1) | 0.909 |
| *MLL*-fusion gene  Positive, n (%)  Negative, n (%) | 17 (4.9)  333 (95.1) | 2 (2.5)  78 (97.5) | 8 (4.5)  171 (95.5) | 2 (3.9)  49 (96.1) | 1 |
| *MLL-*PTD  Mutated, n (%)  Unmutated, n (%) | 22 (6.3)  328 (93.7) | 10 (12.5)  70 (87.5) | 5 (2.8)  174 (97.2) | 4 (7.8)  47 (92.2) | 0.218 |
| *NPM1*  Mutated, n (%)  Unmutated, n (%) | 68 (19.4)  282 (80.6) | 9 (11.3)  71 (88.8) | 42 (23.5)  137 (76.5) | 10 (19.6)  41 (80.4) | 0.561 |
| *C-KIT*  Mutated, n (%)  Unmutated, n (%) | 40 (11.6)  304 (88.4) | 10 (12.5)  70 (87.5) | 26 (14.9)  148 (85.1) | 2 (4)  48 (96) | 0.039 |
| *N-RAS*  Mutated, n (%)  Unmutated, n (%) | 58 (16.6)  292 (83.4) | 10 (12.5)  70 (87.5) | 34 (19)  145 (81) | 5 (9.8)  46 (90.2) | 0.123 |
| Biallelic *CEBPA*  Mutated, n (%)  Unmutated, n (%) | 64 (18.3)  286 (81.7) | 7 (8.8)  73 (91.3) | 35 (19.6)  144 (80.4) | 17 (33.3)  34 (66.7) | 0.038 |
| *DNMT3A*  Mutated, n (%)  Unmutated, n (%) | 48 (13.7)  302 (86.3) | 17 (21.3)  63 (78.8) | 24 (13.4)  155 (86.6) | 3 (5.9)  48 (94.1) | 0.141 |
| *IDH1*  Mutated (%)  Unmutated, n (%) | 25 (7.3)  318 (92.7) | 6 (7.5)  74 (92.5) | 11 (6.4)  161 (93.6) | 5 (9.8)  46 (90.2) | 0.603 |
| *IDH2*  Mutated, n (%)  Unmutated, n (%) | 33 (9.6)  312 (90.4) | 9 (11.3)  71 (88.8) | 17 (9.8)  157 (90.2) | 4 (7.8)  47 (92.2) | 0.887 |
| Favorable^a^, n (%) | 182 (100) | 21 (26.3) | 109 (60.9) | 34 (66.7) | 0.453 |
| Intermediate^b^, n (%) | 125 (100) | 42 (52.5) | 55 (30.7) | 14 (27.5) | 0.653 |
| Adverse^c^, n (%) | 43 (100) | 17 (21.3) | 15 (8.4) | 3 (5.9) | 0.772 |

Abbreviations: NR, no remission; PR, partial remission; CR, complete remission; CRi, complete remission with incomplete hematologic recovery.

^a^Favorable gene abnormalities including *AML1-ETO* fusion genes, *CBFβ-MYH11* fusion genes, mutated *NPM1* without *FLT3-*ITD, and biallelic mutated *CEBPA*.

^b^Intermediate gene abnormalities including mutated *NPM1* with *FLT3-*ITD, wild type *NPM1* without *FLT3-*ITD, *MLL-AF9* and other abnormalities not classified as favorable or adverse.

^c^Adverse gene abnormalities including *MLL*-fusion genes except for *MLL-AF9*, and wild type *NPM1* with *FLT3-*ITD.

**Supplementary Table 3.** Relationship between MRD and peripheral blood recovery

| Factor | Total  (N=196) | CR  (N=154) | CRi  (N=42) | P value  (CR vs. CRi) |
| --- | --- | --- | --- | --- |
| MRD level, %  Median  Range | 0.02  0-3.5 | 0.017  0-2.8 | 0.046  0-3.5 | 0.007 |
| MRD status  Positive, n (%)  Negative, n (%) | 54 (27.6)  142 (72.4) | 35 (22.7)  119 (77.3) | 19 (45.2)  23 (54.8) | 0.004 |

Abbreviations: CR, complete remission; CRi, complete remission with incomplete hematologic recovery; MRD, minimal residual disease.

**Supplementary Table 4.** Relationship between biallelic *CEBPA* mutations and platelet count at diagnosis

| Biallelic *CEBPA* | Mutated, N=64 | Unmutated*,* N=286 | P value |
| --- | --- | --- | --- |
| Platelet count, × 10^9^/L |  |  | P<0.001 |
| Median | 24 | 49 |  |
| Range | 5-199 | 2-1726 |  |
